# Supplementary material for: Complexome profiling of the Chlamydomonas psb28 mutant reveals TEF5 as an early PSII assembly factor
Source: Plant Cell. 2025 Mar 17;37(6):koaf055. doi: 10.1093/plcell/koaf055 (PMC12164590; doi:10.1093/plcell/koaf055)
Supplement: koaf055_Supplementary_Data [file koaf055_supplementary_data.zip › psb28_interactive.zip!/plots/intensity/qualityPlots/peptides/_Cre04.g221550.t1.2.html]

### Cre04.g221550.t1.2

Trivial name:   
SeqCoverage [median]: 10.30  
Mass: 92.43 kDa  
  
 

| Sequence | MissedCleavage | Mass [Da] | wtIntensity | muIntensity | Score |
| --- | --- | --- | --- | --- | --- |
| AAAAAAAVDRAEGFDGLVSTAGER | 1 | 2275.12 | 1387.84 | 0.00 | 93.58 |
| ALSLQEAAMEAVEAVAAR | 0 | 1828.94 | 687.35 | 0.00 | 69.94 |
| GAPVAAALSAAAR | 0 | 1124.63 | 443.59 | 0.00 | 57.35 |
| GLDQALQEDR | 0 | 1143.55 | 506.77 | 460.21 | 169.41 |
| VLMQVGPK | 0 | 870.50 | 302.33 | 0.00 | 76.85 |
| YHAPAGPAAGAAVSAGR | 0 | 1522.76 | 934.87 | 0.00 | 45.14 |
|  |  |  | 4262.7 | 460.2 |  |
